# Suppression of Alcohol Dependence Using Baclofen: A 2-Year Observational Study of 100 Patients

**DOI:** 10.3389/fpsyt.2012.00103

**Published:** 2012-12-03

**Authors:** Renaud de Beaurepaire

**Affiliations:** ^1^Groupe Hospitalier Paul-GuiraudVillejuif, France

**Keywords:** alcoholism, craving, mental disorder, psychotropic drug

## Abstract

**Aims:** The purpose of this study was to examine the long-term effects of baclofen in a large cohort of alcohol-dependent patients compliant to baclofen treatment. **Methods:** A hundred patients with alcohol dependence, resistant to usual treatments, were treated with escalating doses of baclofen (no superior limit). Alcohol consumption (in grams) and craving for alcohol were assessed before treatment and at 3, 6, 12, and 24 months. Assessments were simply based on patients’ statements. The outcome measure was the consumption of alcohol, rated according to the World Health Organization criteria for risk of chronic harm. **Results:** While all patients were rated “at high risk” at baseline, approximately half of them were rated “at low risk” at 3, 6, 12, and 24 months. The sum of patients who were at “low risk” and at “moderate risk” (improved patients) was 84% at 3 months, 70% at 6 months, 63% at 1 year, and 62% at 2 years. The constancy of improvement over the 2-years was remarkable. The average maximal dose of baclofen taken was 147 mg/day. Ninety-two percentage of patients reported that they experienced the craving-suppressing effect of baclofen. Significant relationships were found between the amount in grams of alcohol taken before treatment and the maximal dose of baclofen required, and between the existence of a mental disorder and a lesser effect of baclofen. **Conclusion:** Baclofen produces an effortless decrease or suppression of alcohol craving when it is prescribed with no superior limit of dose. Potential limitations in the effectiveness of baclofen include the coexistence of a mental disorder, the concomitant use of other psychotropic drugs, a lack of real motivation in patients to stop drinking, and the impossibility to reach the optimal dose of baclofen because of unbearable side-effects (sometimes possibly related to too sharp a protocol of dose escalation).

## Introduction

Converging evidence suggest that the gamma-aminobutyric acid-B receptor agonist baclofen is a promising agent for the treatment of alcoholism. While a low dose (30 mg/day) of baclofen significantly decreases alcohol intake (Addolorato et al., 2000, 2002), high-dose baclofen may promote sustained complete abstinence, as shown in several case reports (Ameisen, 2005; Agabio et al., 2007; Bucknam, 2007; Pastor et al., 2012) and in series of patients (Dore et al., 2011; Rigal et al., 2012). However, a number of patients do not respond optimally to high-dose baclofen. In the Dore et al. and Rigal et al. studies, an insufficient efficacy of baclofen was reported in 40–50% of the cases. Rigal et al. showed that the existence of a mental illness and the concomitant use of psychotropic drugs are significantly associated with a reduced efficacy of baclofen. A reduced efficacy of baclofen may also theoretically be related to a number of other factors (i.e., genes, environment, motivation, enduring habits, severity of alcohol dependence, severity of side-effects, treatment observance), which remain to be investigated. In addition, clinicians use different protocols of treatment, and the optimal protocol of treatment with baclofen in alcoholism is not well established. Studies investigating the long-term effects of baclofen in large cohorts of patients should help to characterize some of the factors affecting the efficacy of baclofen.

The present study is a long-term observational study aiming to record precisely the successes and failures of baclofen treatment in alcohol-dependent patients resistant to usual treatments. A hundred consecutively treated patients, all observant to baclofen treatment, were included and followed over 2 years. The effectiveness of baclofen, its undesirable side-effects, and the number of treatment discontinuations were recorded. Preliminary reports on the present study have been published by Ameisen and de Beaurepaire (2010) (60 patients over 6 months), and by Rigal et al. (2012) (a 1-year retrospective study including 67 of the patients of the present study).

## Methods

Prescriptions started in November 2008, following the publication in France of Olivier Ameisen’s book reporting his experience with baclofen (Ameisen, 2008). One hundred and thirty-two alcohol-dependent patients (DSM-IV criteria) were consecutively seen between November 2008 and August 2009. Among them, 32 were excluded because they were lost before the third month of follow-up (some of them provided no feedback after the first visit, others stopped taking their medication rapidly, generally arguing they could not bear the adverse effects of baclofen, and some acknowledged that they were not motivated to stop drinking). These patients were not included in the follow-up because the purpose of the study is to evaluate precisely the effects of baclofen, and treatment-observant patients are needed to achieve this objective (this is not a study based on the intention-to-treat principle). The follow-up of the 100 remaining patients was used for analysis. The effects of baclofen during the 3 first months were not included in the analyses according to the concept of “grace period.” A “grace period” is an early period in a trial where outcome is not considered in the analysis because the effect of the treatment during this period does not represent the full potential of the drug (FDA, 2006; Falk et al., 2010).

Craving for alcohol and alcohol consumption (in grams) were assessed before treatment and at 3, 6, 12, and 24 months. Assessments were based on patients’ statements (no scales used).

Alcohol craving was assessed by two means: (1) Using a visual analogic scale (VAS, score 0–10), (2) The own clinical impression of the author based on the patient’s statements. Given that the VAS appeared to fluctuate according to the patient’s state of mind, optimistic or pessimistic, and was difficult to analyze in these conditions, the author chose to use his clinical assessment, which provided a clear binary appraisal: yes or no (a change or no change in the patient’s craving). The outcome measure was the consumption of alcohol according to the World Health Organization (WHO) criteria for chronic harm (World Health Organization, 2000): patients were rated “at low risk” (i.e., normal: below 40 g/day for men and below 20 g/day for women) or “at medium risk” (between 41 and 60 g/day for men and between 20 and 40 g/day for women) or “at high risk” (superior to 60 g/day for men and 40 g/day for women). Alcohol consumption recorded at each time-point was the average daily consumption of alcohol (in grams) during the 4 weeks before each visit (before enrollment, all patients were every day heavy drinkers, no one being in remission).

The day of the first visit, patients signed a form establishing their informed consent and stating that they suffered from a treatment resistant alcoholism and that they wished to try baclofen. “Treatment resistant alcoholism” means that all patients have been treated before for their alcoholism, in various ways, including medications, hospitalizations, rehab centers, Alcoholics Anonymous, and psychotherapies, and that these treatments failed. The form provided all information regarding precautions in the use of baclofen, its potential adverse effects, the fact that the prescription was off-label, the protocol (progressive dose increase) with the possible necessity of reaching high doses (patients were told that treatment has to be progressively increased until they feel a sufficient indifference toward alcohol to stop drinking or completely control their alcohol intake), and the fact that the personal physician of the patient would be informed of the prescription. After signing this form, patients were prescribed baclofen (10 mg tablets) using the following protocol: 1/2 tablet on day 1 (morning), 1 tablet on day 2 (1/2 morning, 1/2 noon), 1 + 1/2 tablet on day 3 (1/2 morning, 1/2 noon, and 1/2 evening), the 1 + 1/2 tablet being maintained over the four remaining days of the first week. According to Novartis recommendations of prescription (Vidal, 2008), start with half tablet doses is the appropriate procedure of treatment. During the second week, the treatment was increased by three tablets, up to 45 mg/day (1 + 1/2 tablet three times/day), and to 75 mg during on the third week. Patients were called to a second visit at the end of the third week or during the fourth week. In case of ineffectiveness, the treatment was increased by 30 mg/week during the following weeks. Patients were asked to submit to monthly visits. Monthly visits were maintained and patients encouraged to call if any question or particular event came up. Patients drinking only at certain hours of the day (often only in the evening) were advised to increase the dose one hour before the time they usually drank. Patients were also advised to increase the dose in case of stress or in response to a moment of craving.

At the end of the first visit, patients were informed that they were free to drink as usual, given that baclofen is expected to suppress the motivation to drink. When applicable, patients were asked to follow their medical treatment and other forms of therapies as before. Drivers were asked not to drive during the first weeks of treatment because of the risk of somnolence. No therapeutic intervention (psychological, social, or pharmacological) other than baclofen treatment was initiated or suggested.

All patients provided the name and telephone number of their attending physician, and the physician was always contacted by phone (unless the patient had no physician or refused that the physician be contacted). In many cases, the patient’s physician took over the prescription after a while. In some other cases, the treatment was started by a physician before the first visit, so that the first visit occurred after a period of treatment, in general because the patient’s physician did not want to increase the doses (in this case, the starting day of the treatment corresponded to the moment it was initiated by the physician, and the protocol of treatment differed from that described above). Four patients had started treatment by themselves getting baclofen through internet. As a whole, 24 patients did not use the protocol of dose increase exactly as described above.

The following items were recorded at the first visit: age, sex, body weight, and height [the body mass index (BMI) was calculated], drinking history, amount of daily alcohol consumption (in grams), intensity of craving for alcohol, medical, and psychiatric history, present treatments, concomitant addictions, smoking habits, marital status, and employment. For patients drinking intermittently, the daily amount of alcohol drunk during heavy drinking episodes was kept for analysis. When applicable, a psychiatric diagnosis (other than alcoholism) was established based on present symptoms and the patient’s clinical psychiatric history (DSM-IV criteria). During the following visits, patients were asked to report in detail their consumption of alcohol, their craving for alcohol (similar, decreased, or no longer present), their compliance to treatment (doses of baclofen taken), the side-effects of the treatment, their smoking habits, and the use of other psychotropic drugs.

At 3, 6, 12, and 24 months, the effectiveness of the treatment was rated to fit into one of the three WHO categories: “at low risk,” “at medium risk” and “at high risk.” In the “at low risk” category, patients experienced a suppression of craving, and their complete control over drinking was effortless. In the “at medium risk” category, patients experienced a clear decrease in craving but, for various reasons (in general, too strong an attachment to their drinking habits associated with an incomplete motivation to cease drinking), they were not able to control completely their drinking compulsions. In the “at high risk” category, patients either experienced an insufficient reduction of craving, or, although they experienced a significant decrease in craving, after a period of decrease in drinking, relapsed in their addiction. The risk category was defined according to the control over drinking during the last 4 weeks.

Analysis of relationships between continuous variables was based on Spearman’s *r* correlation with asymptotic confidence intervals based on Fisher’s *Z* transform. Comparisons of means were done using Student *t*-test assuming unequal variance (with Satterthwaite adjustment for degree of freedom), and chi-squared tests were used to assess association between categorical variables in two-way cross-classification table. The association between the binary outcome (at low risk vs. at medium or high risk) and psychiatric history and actual treatment (benzodiazepines and/or psychotropic drugs) was summarized as odds-ratio computed from generalized estimating equation models to account for repeated measurements during follow-up. Three regression models were fitted to available data, using one of the three aforementioned binary predictors in each model. We consider time treated as a numerical variable (3, 6, 12, and 18 months), and gender and age as additional co-factors, assuming an unstructured working correlation matrix. Statistical analyses were done with R 2.15.1 statistical software (R Core Team, 2012).

## Results

Patients included 70 men and 30 women, with an average age of 47 (men 48, women 45, *p* = 0.197).

A hundred patients were followed at 3 months, 97 at 6 months, 92 at 12 months, and 87 at 24 months. Among the 13 lost patients at 24 months, 11 had moved providing no feedback, and two had died (one accidental ingestion of a cleaning product, one false passage – both unlikely to be related to baclofen treatment). The number of patients who discontinued baclofen for whatever reason came to 25 at 6 months, 51 at 12 months, and 58 at 24 months.

A summary of patients’ characteristics, according to their risk status at 3 months, is provided in Table 1. Overall, 51% of the patients had a job, and 53% were currently living within a couple (married or not). No significant relationship was found between these variables and the response to treatment at 3 months [professional activity, χ^2^(1) = 0.49, *p* = 0.482; marital status, χ^2^(1) = 1.21, *p* = 0.270].

**Table 1:** **Demographic and biomedical information on participants 3 months after treatment**.

	Not at risk at 3 months (*N* = 50)	At risk at 3 months (*N* = 50)	Overall (*N* = 100)
Gender (female)	32% (16)	28% (14)	30% (30)
Age (years)	48.7 (10.1)	46.0 (11.3)	47.3 (10.8)
BMI (kg/m^2^)	25.0 (5.9)	25.0 (4.4)	25.0 (5.2)
Smoking	74% (37)	78% (39)	76% (76)
Active	54% (27)	47% (23)	51% (50*)
Married	58% (29)	47% (23)	53% (52*)
Psychiatric disorder	48% (24)	70% (35)	59% (59)
Psychotrope use	54% (27)	74% (37)	64% (64)
Benzodiazepine	48% (24)	64% (32)	56% (56)

*1 missing observation.

Ninety-two patients (92%) reported a decrease in their motivation to drink at one time or another during the follow-up. At baseline, all patients belonged to the WHO “at high risk” category. At 3 months, 50% of the responses were classified as “at low risk,” 34% “at medium risk,” and 16% “at high risk”; at 6 months, the percentages were respectively 52, 18, and 27%, they were 48, 15, and 29% at 1 year, and 50, 12, and 25 at 2 years (percentages refer to the 100 patients included; Table 2). Treatment discontinuation increased over time. At 2 years, 45 patients had stopped their treatment, and this concerned all patients in the “at high risk” group, 10 out of 12 in the “at medium risk” group, and 10 (20%) in the “at low risk” group.

**Table 2:** **Number of patients followed, treatment compliance and categories of patients at each time-point**.

	Baseline	3 months	6 months	1 year	2 years
Number of patients	132	100	97 (lost: 3)	92 (lost: 6, deceased: 2)	87 (lost: 11, deceased: 2)
Compliant with treatment	–	100	75 (lost: 3, TD: 22)	49 (lost: 6, deceased: 2, TD: 43)	42 (lost: 11 deceased: 2, TD: 45)
At low risk	–	50 (TD: 0)	52 (TD: 1)	48 (TD: 7)	50 (TD: 10)
At medium risk	–	34 (TD: 0)	18 (TD: 5)	15 (TD: 10)	12 (TD: 10)
At high risk	132	16 (TD: 0)	27 (TD: 16)	29 (TD: 26)	25 (TD: 25)

*TD: treatment discontinuation*.

### Doses

**Figure 1:**
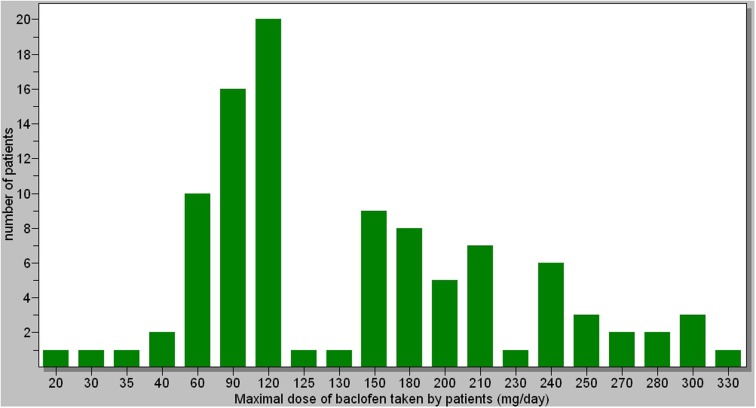
**Maximal dose taken by patients, with the number of patients for each maximal dose**.

The maximal doses taken by patients ranged from 20 to 330 mg (Figure 1). The average dose was 147 mg/day. Most of patients decreased their dose after reaching the maximal dose. There was no significant correlation between the dose and the response to treatment. However, there was a significant relationship between the amount of alcohol (in grams) consumed before treatment and the maximal dose of baclofen needed by patients [*r* = 0.315 (0.127;0.482), *p* = 0.001]. This relationship was significant in both men and women. Twenty patients did not increase the dose of baclofen as much as they should have because they could not tolerate the side-effects above a certain dose (these patients belonged to the “at medium risk” and “at high risk” categories). There were no significant relationships between the maximal doses of baclofen and the BMI of patients patients [*r* = 0.069 (−0.130; 0.262), *p* = 0.498]. The maximal daily dose of baclofen in women was significantly lower than in men (average 127 mg for women vs. 158 mg for men, *p* = 0.025), which may correspond to the fact that the amount of alcohol consumed before treatment was significantly less in women than in men (average daily consumption: 184 g for women vs. 225 g for men, *p* = 0.010).

### Concomitant illnesses and treatments

Fifty-nine percent of patients had one or several concomitant psychiatric disorders: anxiety disorder (53%), depression (34%), bipolar disorder (18%), psychosis (8%), sleep disorder (42%), other addiction (11%, mostly cannabis), eating disorder (5%). The frequency of concomitant psychiatric disorders was significantly [χ^2^(1) = 4.54, *p* = 0.033] higher in females (*n* = 23, 76.7%) compared to men (*n* = 36, 51%). Regarding actual treatments, no significant differences were found between men and women (benzodiazepines, *p* = 0.104; psychotropic drugs, *p* = 0.051).

**Figure 2:**
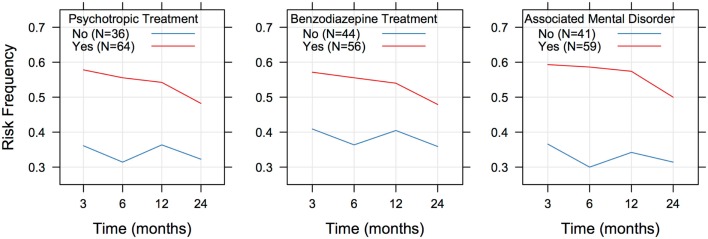
**Risk frequency in individuals depending on concomitant illness and treatments**.

Regression analysis indicated that there was a significant positive association between the existence of a mental disorder (whatever the nature) and an unfavorable outcome [OR = 2.400 (1.515; 3.801), *p* < 0.001]. Likewise, positive associations were found between the outcome and use of benzodiazepines [OR = 1.637 (1.053; 2.543), *p* = 0.028] or psychotropic drugs [OR = 2.064 (1.308; 3.255), *p* < 0.002]. In each case, the probability of being at risk did not evolve significantly with time (Figure 2), and there were no differences between males and females or according to age (Table 3).

**Table 3:** **Parameters estimates for régression models (GEE), assessing for the relation between outcome and (a) psychotrope use, (b) benzodiazepine, and (c) any psychiatric disorders**.

Parameter	Estimate	SE (robust)	Wald *z*	*p*-Value	OR (95% CI)
**A**					
Intercept	0.258	0.560	0.21	0.645	1.294 (0.432; 3.874)
Time	−0.013	0.013	0.97	0.325	0.987 (0.961; 1.013)
Use of psychotrope (yes)	0.725	0.233	9.71	0.002	2.064 (1.308; 3.255)
Gender (female)	0.152	0.240	0.40	0.526	1.165 (0.727; 1.864)
Age	−0.016	0.001	2.60	0.107	0.984 (0.965; 1.003)
**B**					
Intercept	0.411	0.559	0.54	0.462	1.508 (0.504; 4.513)
Time	−0.013	0.013	0.91	0.340	0.987 (0.962; 1.013)
Use of benzodiazepine (yes)	0.493	0.225	4.80	0.028	1.637 (1.053; 2.543)
Gender (female)	0.209	0.238	0.77	0.380	1.233 (0.773; 1.966)
Age	−0.015	0.001	2.43	0.119	0.985 (0.966; 1.004)
**C**					
Intercept	−0.115	0.581	0.04	0.843	0.891 (0.285; 2.781)
Time	−0.014	0.014	1.00	0.318	0.987 (0.961; 1.013)
Psychiatric disorders (yes)	0.875	0.235	13.91	0.000	2.400 (1.515; 3.801)
Gender (female)	0.128	0.238	0.29	0.592	1.136 (0.712; 1.812)
Age	−0.009	0.010	0.76	0.383	0.991 (0.971; 1.011)

*Reference categories are reported in parenthesis for categorical predictors*.

Thirty-two percent of patients had a somatic illness (mostly cardiovascular and hepatic). Seventy-six percent of patients were smokers (Table 1). Among them, nine decreased the number of cigarettes smoked during baclofen treatment (five stated that the decrease was related to baclofen treatment), and eight increased the number of cigarettes smoked.

### Side-effects

Eighty-eight percent of patients reported at least one undesirable side-effect that could possibly be attributable to baclofen. They were always benign. Side-effects, at one moment or another of the treatment, independent of their severity or duration, were the following: substantial fatigue/sleepiness 64%, insomnia 31%, dizziness 21%, paresthesia 18%, nausea/vomiting 17%, sensory alterations 16%, sexual changes (decrease or increase of libido) 15%, various forms of pain 20% (including headache 6%), bowel disorder (diarrhea, constipation) 12%, dysphoria 10%, weight loss 10%, memory loss 9%, hypomania (including one case of clear-cut mania) 7%, change in eating behavior 7%, weight gain 6%, sweat/hot flush 6%, tinnitus 6%, mental confusion 5%, cutaneous eruption 3%, respiratory difficulties 3%, sugar craving 3%, other 19% (which included irritability, transient amaurosis, photosensitization, urine incontinence, cramps, muscular contraction, muscular spasms, trembling, elocution difficulties, hypersialorrhea, water retention/edema, shouting, electric discharges, palpitations). No significant relationship was found between side-effects [globally or individually (when statistically valid)] and treatment response. No significant relationship was found between side-effects and the existence of a concomitant mental disorder.

## Discussion

The results of the present study show that treatment with baclofen at sufficient doses produces a complete and effortless control of alcohol dependence in approximately half of the patients at any given point in time. In addition, 92% of the patients reported an effortless decrease in their motivation to drink. These effects may be interpreted according to two parameters: that of effectiveness (the effect of the treatment on real life behaviors) and that of efficacy (the effect on the motivation itself to drink).

In terms of effectiveness, the highly beneficial effects of baclofen in the present study were observed at 3, 6 months, 1 year, and 2 years. The number of patients rated “at high risk” increased between 3 and 6 months, and then stabilized. After 6 months, the number of patients in each category remained relatively stable. The low rate of relapse after 6 months in abstinent patients is remarkable and must be emphasized. The increase in number of patients rated “at high risk” between 3 and 6 months was due to the fact that a number of patients who reduced their drinking after baclofen initiation (rated “at low risk” or “at medium risk” at 3 months) were unable to maintain a reduced consumption, likely because of too strong an attachment to drinking habits, and to an insufficient motivation to quit drinking. Many patients acknowledged they were ritualized in compulsive drinking and could not stop drinking even though their appetite for alcohol was reduced by baclofen. Conversely, many patients needed long periods of time to go from the categories “at medium risk” or “at high risk” to the category “at low risk.” These patients clearly experienced the beneficial effects of baclofen, but had to make strong efforts to give up their drinking rituals and their attachment to alcohol. The strength of the willingness to stop drinking may differentiate those who relapsed after a period of improvement from those who progressively improved to reach a complete control over drinking. Baclofen should be considered a major help for drinking cessation, but other factors (psychological and environmental) are likely to play an important role with many patients. Patients who rapidly succeeded in acquiring complete control over drinking (during the first weeks or months) said that they effortlessly reached a state of indifference toward drinking (a concept introduced by Ameisen, 2005). This concerned approximately 50% of the patients. Some patients who had stopped drinking were able to stop baclofen after a long period of treatment, and did not relapse (1 patient at 6 months, 7 at 1 year, and 10 at 2 years – however, several of these patients acknowledged they always kept with them some tablets of baclofen in case of a sudden craving). Other patients who had stopped drinking started to experience craving for alcohol again when they lowered baclofen beneath a certain dose. No element (BMI, sex, social, family, and professional features), with the exception of the amount of alcohol consumed before treatment, had a predictive value regarding the dose of baclofen needed, even though the inter-individual variability was very important. Given that the usual pharmacological treatments of alcoholism have a limited long-term effectiveness (Johnson, 2008), and although the present study was only observational, it adds new evidence demonstrating that baclofen is a treatment of major interest for alcoholism. It should also be noted that, in addition to the patients abstinent at the different time-points, the patients who went from the “high risk” category to the “moderate risk” category were also markedly improved, providing a total of 84% of improvements at 3 months, 70% at 6 months, 63% at 1 year, and 62% at 2 years.

The outcome into the second year of all patients who had achieved improvement at 1 year shows a remarkable stability. Of the 48 patients belonging to the “low risk” category at 1 year, 46 were rated in the same category at 2 years. The two patients who relapsed had episodes of heavy drinking and periods of recovery during the second year, they maintained baclofen treatment, and were categorized “at medium risk” at 2 years. Three patients rated “at medium risk” and one “at high risk” at 1 year improved and were rated “at low risk” at 2 years. These cases illustrate the great difficulties of some patients to become abstinent despite the anti-craving effects of baclofen. Among the patients rated “at low risk” at 2 years, all were not constantly sober during the year, many (about one third) had moments of short duration relapses, generally occurring in response of stressful events or situations, but these short relapses were not sufficient to justify a change of category. At 2 years, I continue to personally follow 19 of the “at low risk” patients, others are followed by their attending physician. Besides the fact that 10 patients of the “at low risk” group had stopped baclofen treatment at 2 years, the majority of 40 remaining patients (31/40) had considerably reduced baclofen: 167.7 mg/day average maximal dose during the follow-up, 107.4 mg/day average dose at 2 years (nine patients remained on their maximal dose).

In terms of efficacy, all patients except eight reported that baclofen reduced or completely stopped their motivation to drink. The analysis of the eight cases of patients who reported no effects of baclofen on craving shows that four of them were very uncomfortable with baclofen-induced side-effects, and probably did not reach sufficient doses. For the four remaining patients (two of them reached doses above 280 mg), the question of an insensitivity to baclofen must be raised. Alternative explanations could be a denial of the effects of baclofen or a poor compliance with treatment. Further work is necessary to determine whether certain individuals are really insensitive to the anti-craving effects of baclofen. Recently, Addolorato et al. (2011) showed a dose-response effect of baclofen in the treatment of alcohol dependence. There was no dose-response effect in the present study, but the present study is not in contradiction with the Addolorato et al. study. The goal of the present study was to increase the doses of baclofen until the achievement of a complete abstinence, while the goal of the Addolorato et al. study was to analyze (number of drinks per day) the effect of two given doses (30 and 60 mg) during a given period of time. In fact, in the present study, many patients decreased progressively their drinking, starting to drink less at low doses, but the doses were nevertheless increased with the aim of reaching abstinence.

The occurrence of side-effects limited the effectiveness of baclofen in certain patients. Eleven patients said that they discontinued treatment because they could not tolerate the side-effects, and 20 patients did not reach an efficacious dose because of the worsening of side-effects when doses were increased. Even though a number of patients said they could not tolerate the side-effects of baclofen, these side-effects were always benign. Side-effects appear at a certain dose during the dose escalation. Dose escalation may have been too sharp for certain patients. Slower dose escalation should be considered.

A fairly high number of patients in the present study presented with comorbid mental disorders. A significant relationship was found between the existence of a mental disorder and an unfavorable outcome. The relationship was significant at 3 and 6 months, but not at 1 and 2 years. Of the 50 “at low risk” patients at 2 years, 26 had a comorbid mental disorder, and 24 did not. Therefore, according to the present study, the existence of a mental disorder may compromise improvement at 3 and 6 months, but not at 2 years. In other words, the coexistence of a mental disorder may delay the effectiveness of baclofen. Alcohol dependence is known to be frequently associated with mental disorders, and these disorders, in particular depression, are known to hamper the effectiveness of treatments (Pettinati, 2004). In the present study, no significant relationship was found between a less satisfactory outcome and a particular category of pathology. However, there was a significant relationship between the use of psychotropic drugs, particularly benzodiazepines, and a worse outcome. Further work is needed to determine whether the effect of baclofen could be dampened by a concomitant treatment with certain psychotropic drugs, particularly benzodiazepines. It should also be remembered that people with mental disorders often show a bad compliance with treatments (Wilder et al., 2010), and some patients in this category may have been poorly compliant with baclofen in the present study.

In conclusion, the results of the present study show that baclofen is very effective in the treatment of alcohol dependence, and, in particular, extremely efficacious in effortlessly reducing motivation to drink. High doses of baclofen were often necessary, but not always, to obtain an optimal effect. The side-effects of baclofen, the co-occurrence of psychiatric illnesses (possibly the use of concomitant psychotropic medications), and the absence of a strong willingness in some patients to stop drinking, appear to be the principal limitations to the effectiveness of baclofen. The possibility that a slowing in dose escalation can reduce side-effects should be considered.

## Conflict of Interest Statement

The author declares that the research was conducted in the absence of any commercial or financial relationships that could be construed as a potential conflict of interest.
